# Supplementary material for: Impact of Manganese on Neuronal Function: An Exploratory Multi-Omics Study on Ferroalloy Workers in Brescia, Italy
Source: Brain Sci. 2025 Jul 31;15(8):829. doi: 10.3390/brainsci15080829 (PMC12384076; doi:10.3390/brainsci15080829)
Supplement: Supplementary file 1 [file brainsci-15-00829-s001.zip › brainsci-3712284-supplementary.pdf]

Supplementary Data

Table S1. The set of metabolites with significantly biological (FC > 1.5) and statistical ($p < 0.05$) differences from volcano plot analysis in the student's t-test. Significantly upregulated metabolites (total=49) in the plasma of Mn exposed workers compared to control group, arranged in descending order from the highest to lowest fold change (FC). B. Significantly downregulated metabolites (total=13) in the plasma of Mn exposed workers compared to control group, arranged in descending order from the highest to lowest fold change (FC).

(a)

Upregulated Metabolite	FC	p value
Oxymesterone	5.844	0.025143
HEPES	5.561	0.04226
diacetyl trimer	5.031	0.024803
Palmitoyl glutamic acid	3.4201	0.013744
Guanidinosuccinic acid	3.174	0.016216
LIC9QAS59U	2.778	0.0053104
2-Hydroxy-2-methylbutyric acid	2.774	0.031764
Tetrahydrofuran	2.774	0.031764
C6 H6 N2 O2	2.7091	0.031643
Butyric acid	2.6534	0.0059909
2-Aminoisobutyric acid	2.5645	0.014392
(-)-pinellic acid	2.5033	0.0089942
C20 H37 N O4 S	2.5013	0.046599
C7 H6 O6 S	2.4234	0.015619
1-O-palmitoyl-2-O-arachidonoyl-sn-glycero-3-phosphocholine	2.157	0.015904
L-(2,3-~2~H_2_)Threonine	2.116	0.040442
(S)-(+)-N-Boc-3-pyrrolidinol	2.1056	0.044566
2-Deoxyhexopyranose	1.9648	0.024223
D-Mannoheptulose	1.9648	0.024223
(E)-Aconitic Acid	1.9499	0.038327
gamma-Glu-Ile	1.9329	0.014855
Purine	1.8922	0.025603
2-Furoic acid	1.8862	0.034909
(±)5-iPF2?-VI	1.8752	0.047639
2-Butoxyacetic acid	1.8526	0.019614
C5 H6 O4	1.843	0.026972

Upregulated Metabolite	FC	p value
Choline	1.8248	0.014724
Pellitorin	1.8167	0.018208
4-Octylphenol	1.8167	0.018208
ACETYL PROLINE	1.7999	0.012912
AMPA	1.7595	0.0064805
Ethylmalonic acid	1.7443	0.0041374
N-[(3S)-2-Oxotetrahydro-3-furanyl]pentanamide	1.7305	0.0037919
L-gamma-Glutamyl-L-valine	1.7125	0.028958
2-(Methylamino)isobutyric acid	1.6802	0.021439
2-Aminoethyl (2R)-3-[(1Z)-1-hexadecen-1-yloxy]-2-hydroxypropyl hydrogen phosphate	1.6618	0.003523
Hexacosanedioic acid	1.6338	0.022585
Trinonanoin	1.6199	0.031432
Homovanillic acid	1.6157	0.035027
(2S)-3-{[(1Z)-11-Methyl-1-dodecen-3-yn-1-yl]oxy}-1,2-propanediol	1.583	0.015553
PAF C-18-d4	1.5824	0.014784
C20 H26 O	1.5756	0.032239
L(-)-Carnitine	1.5749	0.015076
DAz-1	1.5501	0.0061043
Y-L-Glutamyl-L-glutamic acid	1.5401	0.0046488
Glu-Glu	1.5356	0.0070337
dilauroyl peroxide	1.5213	0.041073
C11 H16 N4 O5	1.5206	0.01287
(5Z,8Z,11Z,14Z)-N-[2-(3,4-Dihydroxyphenyl)ethyl](5,6,8,9,11,12,14,15-~2~H_8_)-5,8,11,14-icosatetraenamide	1.5188	0.0019601

(b)

Downregulated Metabolite	FC	P value
4-Allyl-2-hydroxy-6-methoxyphenyl hydrogen sulfate	0.06805	0.031975
C19 H37 N O4	0.11713	0.031942
(2S,3aR,4R,5S,6R,6aS,7R,9aS,10aR)-9a-(Acetoxymethyl)-2,6,7-trihydroxy-7-isopropyl-4-methyl-1-methylenetetradecahydrodicyclopenta[a, d][8]annulen-5-yl acetate	0.24557	0.000174
Sacubitril	0.28154	0.004546
C6 H12 O6	0.28408	0.013986
C7 H8 O5 S	0.29533	0.03716
C6 H10 N3 O4 P	0.36203	0.022048
(2Z,6E)-N-(2-Hydroxyethyl)-2-methyl-2,6-icosadienamide	0.48614	0.020388
16NS	0.50839	0.025294
3,4-Dimethoxyphenethylamine	0.5568	0.044327
(3S)-3-{(Z)-[(3S)-3-{(Z)-[(3R)-3-Amino-1-hydroxy-4-methylpentylidene]amino}-1-hydroxybutylidene]amino}-5-methylhexanoic acid	0.56918	0.044344
Methyl 3-{(2S,3R)-3-[(2Z,5Z,8Z,11Z,14Z)-2,5,8,11,14-heptadecapentaen-1-yl]-2-oxiranyl}propanoate	0.59735	0.024519
N-[(2S,3R,4E,6E)-1-(beta-D-Glucopyranosyloxy)-3-hydroxy-4,6-pentadecadien-2-yl]octadecanamide	0.60556	0.025875

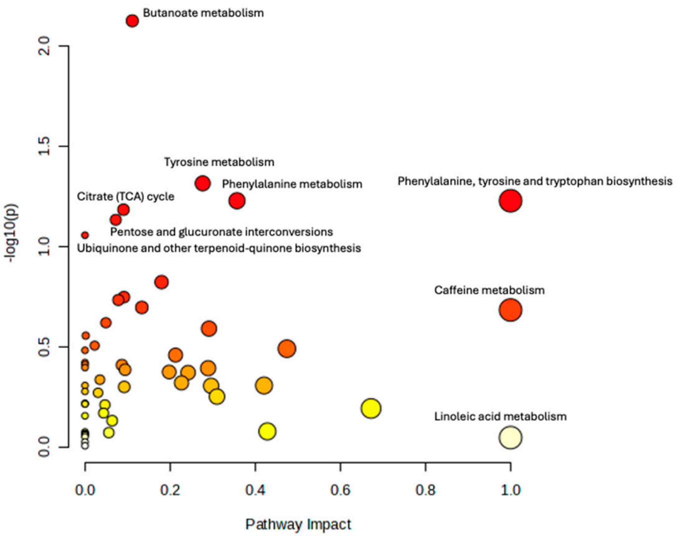

Figure S1. An overview of Pathway Analysis using distinct metabolites identified through a student's t-test was conducted by global test using Homo sapiens KEGG library. The X-axis indicating the impact or importance of respective pathways on Mn exposure study. The Y-axis describe the statistical significance of results (p-value). The size of bubble showing the number of compounds involved in the pathway that are affected by the exposure. Larger bubbles mean greater number of compounds. The bubble color represents the magnitude of p-value, a more intense red color indicates a lower p-value.

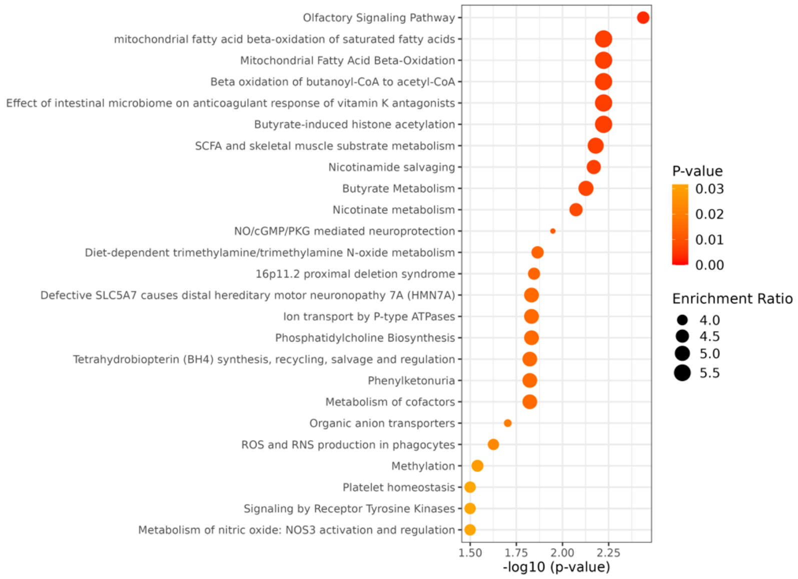

Figure S2. Dot plot of Metabolomic Enrichment Analysis using RaMP-DB as a reference. It indicates the top 25 enriched metabolite set in the study of metabolite profile of Mn-exposed workers than controls. The X-axis showing the p-value and Y-axis indicating metabolites set.

Table S2. Significantly Enriched Metabolic Pathways Based on Differential Metabolite Analysis. Total: The total number of metabolites in each set. **Hits**: The metabolites in the set that are significantly changed in the experimental data. **P Value**: The probability that the observed number of hits (or more extreme) would occur by random chance, given the total number of metabolites tested.

Metabolite Set	Hit	Total	Hit#	P value
Olfactory Signaling Pathway	Lactic acid, Propionic acid, Geranyl acetate, Butyric acid	56	4	0.0036546
mitochondrial fatty acid beta-oxidation of saturated fatty acids	Butyric acid	39	1	0.0059909
Mitochondrial Fatty Acid Beta-Oxidation	Butyric acid	63	1	0.0059909
Beta oxidation of butanoyl-CoA to acetyl-CoA	Butyric acid	13	1	0.0059909
Effect of intestinal microbiome on anticoagulant response of vitamin K antagonists	Butyric acid	18	1	0.0059909
Butyrate-induced histone acetylation	Butyric acid	4	1	0.0059909
SCFA and skeletal muscle substrate metabolism	Butyric acid, Propionic acid	4	2	0.0066115
Nicotinamide salvaging	Lactic acid, Propionic acid, Butyric acid	45	3	0.0067723
Butyrate Metabolism	Butyric acid, Acetoacetic acid	14	2	0.0074634
Nicotinate metabolism	Lactic acid, Propionic acid, Butyric acid, Glutamic acid	56	4	0.0084484
NO/cGMP/PKG mediated neuroprotection	Glutamic acid, Citrulline	15	2	0.011276

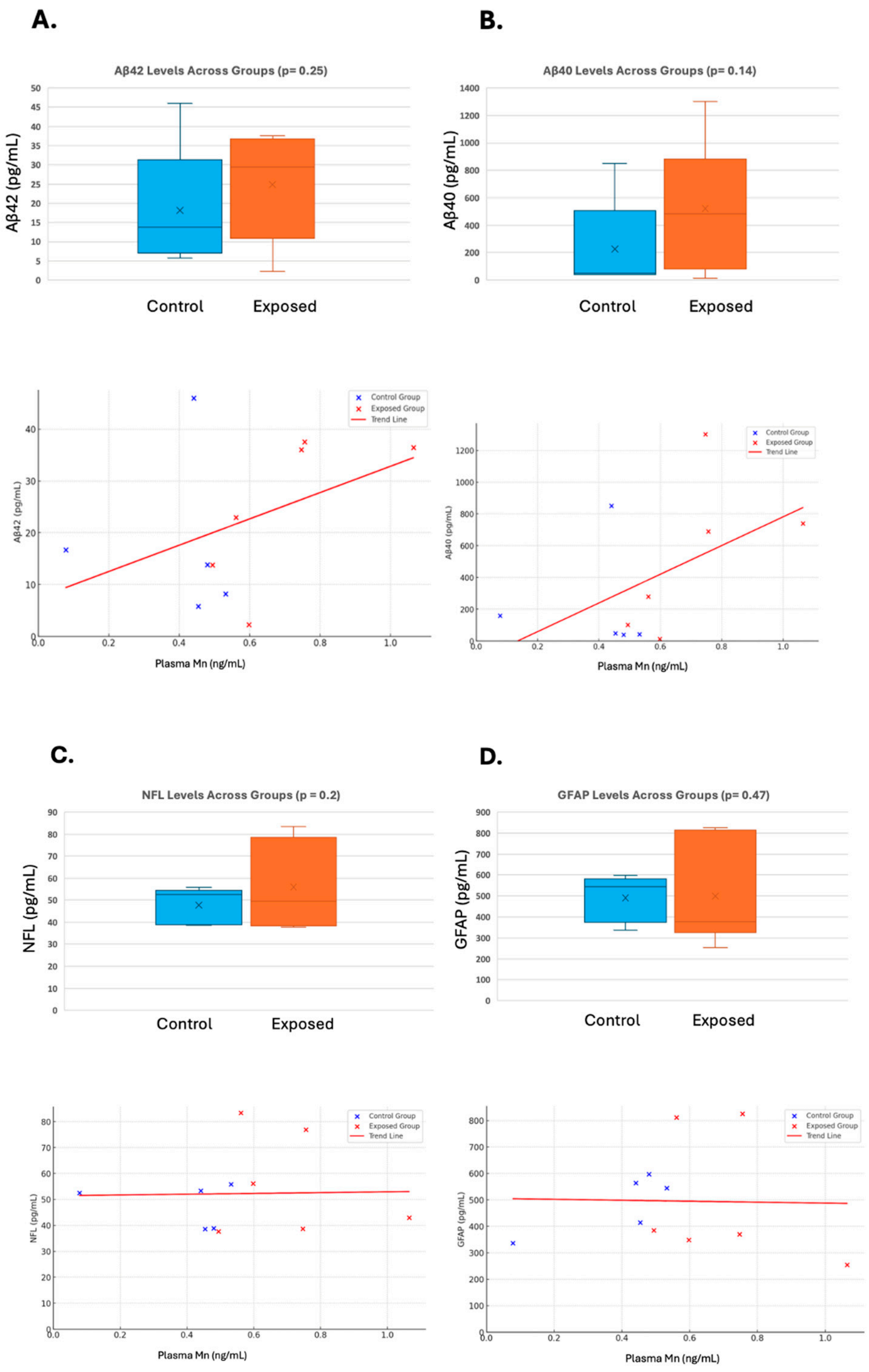

Figure S3. Comparative level of AD biomarker proteins. (a) Ab42 (b) Ab40 (c) NfL (d) GFAP Up**:** Bar chart of comparative level of proteins in plasma samples of Mn exposed workers and control group measured using single molecule arrays (Simoa) in Quanterix SR-X platform. Down: The Scatter plot represents the individual data points for proteins level versus PMn values. Blue points represent control individuals, while red points represent Mn exposed individuals. The red line indicating collective trend for both Mn exposed and control groups.

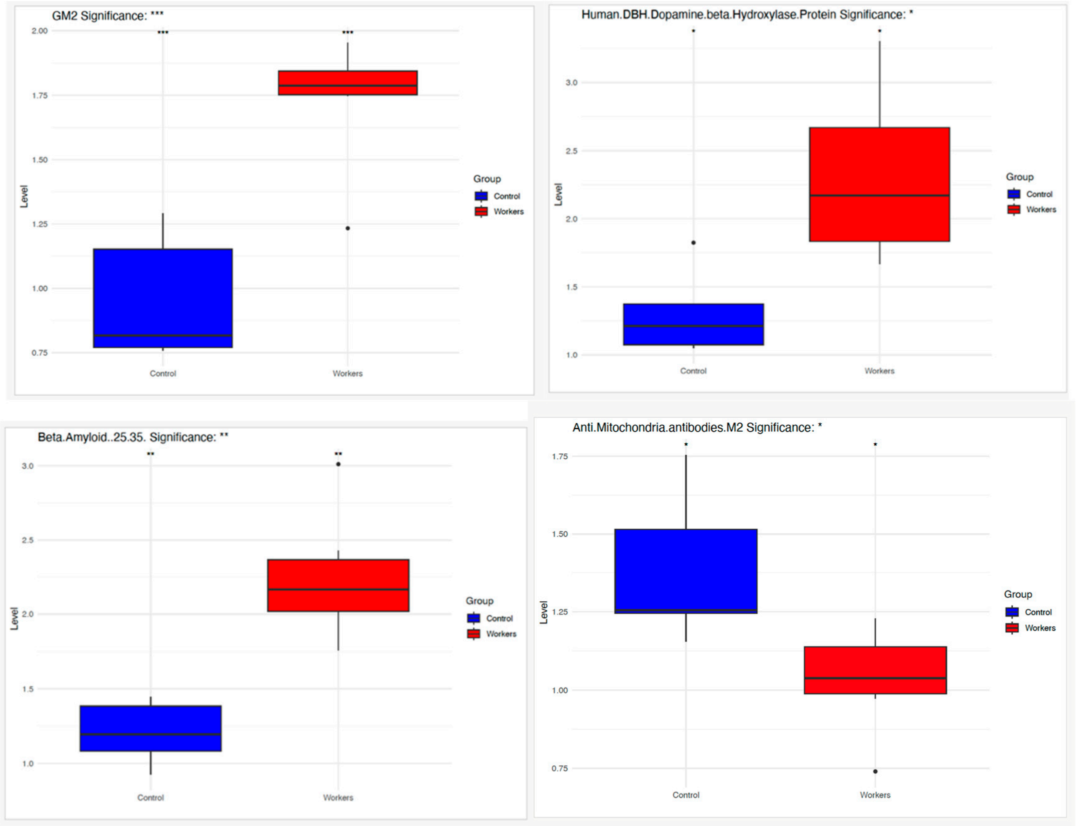

Figure S4. Bar plots of significantly distinct GM2 and dopamine beta-hydroxylase (DBH), Beta amyloid 25-35 and AMA-M2 antibodies. The antibodies pertinent to neurology exhibited significantly varied abundance levels ($p < 0.05$) in the plasma samples of the Mn-exposed group compared to controls, as assessed through protein chip analysis.

Table S3. The panel of antigens employed in protein microarray analysis to assess plasma autoimmune antibody levels among Mn-exposed workers and control subjects.

AutoImmune Antigen	3000/2675	3001/2849	3002	3003	3004/4755	3005/5608	3006/5965	3007/6013	3008/5997	3009/6069	3010/6166
Group	Workers	Workers	Workers	Workers	Workers	Workers	Control	Control	Control	Control	Control
GM2	1.74613902	1.23335208	1.85631216	1.95385619	1.80451141	1.76957842	1.29186642	1.15181285	0.770845	0.81667917	0.758345
Beta-Amyloid (25-35)	1.75635799	2.42920375	2.16777418	3.01151218	1.97099894	2.16365473	1.08168179	1.44637487	1.38335458	1.19585167	0.92501417
Tubulin Beta (TUBb)	1.84831955	2.00003042	2.5	2.5290149	2.88130944	3.31325265	1.82843457	2.08081638	1.45835583	1.18335125	1.31668667
5-Hydroxytryptamine (5-HT)	2.35808348	5.91259167	5.36544956	4.24372838	3.66272877	5.8659647	3.34073924	1.83345946	2.10419875	1.62919167	2.65420708
Human DBH/Dopamine beta-Hydroxylase Protein	1.79609397	1.66669208	1.94352179	2.39563807	2.76047347	3.30321264	1.37388963	1.82401852	1.21251875	1.07501667	1.05001625
Human NOVA1 Protein	1.8358311	1.8958625	2.41279078	2.36568673	2.39795913	3.04969994	1.85406815	1.36329323	1.51252333	1.80419417	1.67085875
Human Siglec-1 / CD169 Protein	2.15485887	3.02921292	4.33554836	3.45797434	2.93233175	3.58433787	2.93062237	1.91465197	1.88752875	1.95003	1.21251875
Recombinant Adaptor Related Protein Complex 3 Beta 2 (AP3b2)	2.02202486	1.98336375	1.92691034	1.85885438	2.55907644	2.16616473	1.38926892	2.16389858	1.27918625	0.86668	1.08335
Anti Mitochondria antibodies M2	0.97269636	1.170018	1.04126677	1.22762151	0.74001137	1.03501587	1.51502319	1.255019	1.1550175	1.245019	1.75400625
Human Brain Cerebellum Protein	1.25681188	1.36252083	2.03073101	1.17512142	2.33351247	1.45080316	1.35167499	1.11216031	0.89168042	0.975015	0.88751375
Recombinant Human GFAP Protein	1.53496835	1.48335583	1.87707638	1.78491176	2.20730414	2.36194789	1.94292533	1.42749279	1.16251792	1.0416825	1.09168333
Recombinant Human Amphiphysin/AMPH Protein	1.96071677	2.67504083	5.0830577	2.01656676	2.72287737	3.45883527	1.23376648	2.33761386	1.39168792	1.98753042	1.30835333
rhCarbonic Anhydrase VIII	3.24477733	2.7750425	4.65116231	2.98109286	3.61976488	3.81275055	3.09637859	2.65105701	2.77920917	2.74170875	1.10835042
Recombinant Leucine-rich glioma-inactivated protein 1 (LGI1)	2.70322347	2.1375325	1.76079743	1.18214163	1.78571444	1.75702816	1.28503109	2.03549795	1.00834875	0.88751375	0.84167958
Recombinant Human RAGE Protein	2.10830986	1.42918833	1.71511623	1.33470621	1.11439305	1.566265	0.73479175	1.73904869	0.87084667	0.98334833	0.95001458
PDC-E2	1.2627986	1.190018	0.74616138	0.97186704	0.76501175	1.19501831	1.33002037	1.1950185	1.040016	1.31002	1.72369012
PM/Scl75	0.88737215	1.045016	2.42802203	1.35976132	0.78501205	1.11001701	1.67502564	1.8000275	1.2600195	2.9000445	2.6808146
Human STK23/MSSK1/SRPK3/SPRY3 recombinant protein	2.36262474	2.26253458	3.90780739	2.73633435	1.57626221	1.89759052	2.08304967	1.5143508	1.97503	1.95836333	1.2458525
Ribosomal P1	1.11774748	1.100017	1.07005759	1.4620631	0.76001167	1.13001732	1.49002281	1.4650225	1.240019	1.845028	1.02208754
U1-snRNP C	4.54778056	1.6500255	6.64347364	6.25319679	0.85001305	2.10003212	2.07003166	1.9200295	1.0750165	1.910029	1.50714599
Human GRM1 / MGLUR1 Protein	1.65077151	1.71252625	1.47425239	1.78023194	3.40494001	2.67319214	1.81134665	2.03738614	1.33335375	0.92918083	1.14168417
Recombinant Bone Morphogenetic Protein 1 (BMP1)	3.09037236	1.9208625	1.85631216	0.52087229	3.47475903	2.02058237	1.48325401	1.46336876	1.47085583	0.92501417	1.29168625
Ribosomal P2	1.14334479	1.0800165	1.15643008	1.08695655	0.7550116	1.06501633	1.53002342	1.5350235	0.9950155	2.3900365	1.16067573
POLY(L-GLUTAMIC ACID)	1.33742031	1.65835875	1.59883728	1.02957705	1.726638	2.24146579	1.42686303	1.16125398	1.17918458	0.88751375	1.60835792
Recombinant Human NARG2 Protein (NMDA)	5.26907282	4.2959	5.71843836	2.98717663	4.31525373	5.10793159	2.15652772	6.02152724	3.66255583	1.78336042	1.80419417
U1-snRNP A	3.37030746	2.7250415	8.64203484	5.2344415	0.74501144	1.19501831	2.35503601	1.565024	1.31002	1.820028	0.9874406
Anti angiotensin II type1 receptor	1.62969276	1.1500175	1.51871415	1.34271102	0.80001228	1.0700164	1.84502823	1.9950305	1.105017	1.2200185	2.70246866
Histidyl-tRNA Synthetase(Jo-1)	21.8088743	6.080095	22.1473137	13.4782605	1.20001839	2.36003609	8.48012947	4.985076	1.4800225	4.115063	4.20528384
Recombinant Human GAD2/GAD65 Protein	3.12897303	3.14171458	1.8978406	3.25346279	4.59452176	5.01254901	2.5	4.78096819	1.30418667	0.97918167	1.61252458
Recombinant Human Enolase 2/Neuron-specific Enolase Ptotein	4.02247876	4.4709	6.96428726	4.05934053	3.44253611	3.05973995	2.8827762	3.20052945	4.6667375	2.01253083	3.57922125
Recombinant Human Dopamine D2R/DRD2	2.51930034	3.75422417	3.4260797	3.90256411	2.98066614	2.07831316	1.33629552	4.24848945	2.25003458	1.51252333	1.30418667
Human ELAVL3 / HUC Protein	3.48319667	2.46670417	3.77491698	3.62457839	2.05692808	1.61646579	2.05912488	3.05136063	2.52087167	1.61252458	1.44168875
Formiminotransferase Cyclodeaminase (LC1)	7.12884194	3.1100475	7.54078723	4.47570324	1.14501755	1.73502655	3.71005669	3.150048	1.4600225	1.250019	2.37332209
Recombinant GRB2 Related Adaptor Protein 2 (GRAP2)	3.31743856	2.67087417	1.83139518	1.95993996	2.93233175	9.22439738	1.35680137	3.73489449	1.74586	1.23751875	1.07918333
U1-snRNP B/B'	2.72184336	1.2800195	6.12763937	5.75447558	0.68501053	1.29501984	2.2100338	1.690026	0.860013	1.2650195	1.2906023
Recombinant Human NRXN3 Protein	2.5	3.32088417	4.70930095	4.18008185	2.75778823	2.93674735	2.90840808	3.57439653	1.96253	3.27921667	1.408355
Human Tau-441 / 2N4R Protein	6.41008041	5.02090833	4.99999834	4.70656853	2.68528396	4.6234937	3.7491456	4.37311181	4.62090417	4.2334	1.56669083
CENPB	1.20733784	1.895029	2.68713996	1.39386191	0.86501328	1.38502121	2.19003349	1.96503	1.255019	2.5050385	4.96319004
Fibulin	1.75767929	1.715026	1.40595027	2.42540493	1.20001839	2.49503815	4.70507187	2.3300355	1.4050215	2.7750425	2.27804246
Recombinant Human Titin protein	2.54541256	2.27503458	4.52242673	2.8818792	2.53759395	10.4216858	3.18352697	2.39614772	1.93752958	1.35002083	2.52920542
Recombinant Human AIF-1/Iba1 Protein	3.30267834	3.95006042	5.20764051	3.87588865	2.65843179	3.36596269	4.01913984	2.65483339	3.55005417	3.06671333	1.27501958
Recombinant Calcium Channel Alpha 1B Subunit (CACNa1B)	4.93528483	25.7003917	23.1021594	4.81233721	4.70461944	6.90010052	4.90430766	6.06117921	10.404325	3.25838292	3.57922125
Recombinant Human MBP Protein	3.54677539	3.29588375	5.2200988	3.23895534	1.37218047	3.29568264	3.40909091	3.24962236	1.96669667	2.52087167	1.08335
Nucleolin	10.9556309	4.000061	4.42178484	36.2787713	1.38002113	2.39503662	3.84005867	3.080047	1.575024	2.0550315	1.99653526
Proliferating Cell Nuclear Antigen	1.11774748	1.0800165	2.05374294	1.15089517	0.84001289	1.19001823	1.62502487	1.5450235	0.985015	2.0650315	1.49848437
SmD2	5.07252513	4.4250675	11.1708255	17.7195221	1.03501587	2.22003395	6.32009651	4.435068	1.5850245	1.900029	2.8757038
Nup62	2.35068263	1.6600255	1.30518237	1.50042627	0.81001244	1.32502029	6.08009285	1.570024	1.29002	1.450022	3.13555693
TIF1 gamma	0.93856676	0.8900135	2.1353168	1.17220804	0.78001198	1.06501633	1.44502213	1.6550255	0.835013	1.7200265	5.04114468
HLA–B*14:02	2.242535	2.33949522	1.91335713	1.71365543	2.0179372	2.00607155	1.81306453	1.93687583	1.85334155	2.00328195	2.0062124
Cluster of differentiation 11b (CD11b)	2.46593945	1.86252833	1.66528227	1.54483308	2.05692808	2.86646479	2.22146352	2.61706961	1.25418583	0.93751417	1.10001667
GP2	2.32935147	1.575024	7.34165158	2.67263425	7.15010918	1.52002327	2.26003456	2.105032	1.0750165	2.365036	4.03637952
Recombinant Human CD68/SR-D1 Protein	2.75544905	1.75419333	2.75747489	2.98951654	1.16272824	1.29769079	0.87662352	2.41691968	2.44170417	1.37085417	0.65834333
U1-snRNP 68/70 kDa	4.45819149	5.21508	9.73848407	6.31287284	0.93001427	3.71505676	7.73011803	2.095032	2.2450345	2.4450375	1.63274175
Recombinant Human Recoverin Protein	3.96003536	3.56255458	5.75996596	3.37981989	2.07572505	2.14859447	2.76828544	3.63481858	3.47088625	2.64587375	0.92918083
Recombinant Human S100B	3.73410447	66.226	4.64285679	6.88459106	2.8974209	2.85391478	3.46377396	3.06835433	3.48338667	2.97921208	1.63335833
Proteinase 3	3.32764514	1.170018	3.01583457	1.4791134	0.80501236	1.20001839	1.37502106	1.2000185	1.0150155	1.6100245	1.55911662
RNA polymerase III	3.22525634	1.710026	4.89923198	2.78346119	0.90001381	1.10501694	4.98507614	2.0050305	1.165018	3.675056	21.9055873
BPI	3.70307201	1.2850195	4.89443359	13.2011932	0.79001213	1.09001671	1.58002419	1.055016	0.9300145	1.8550285	4.66868607
DFS70	11.3865203	11.05517	7.34884917	4.03665808	0.9650148	3.63505554	5.14507858	2.8450435	2.6450405	5.82009	5.99826741
Gp210	2.78157026	1.120017	1.0892514	1.21483379	0.78001198	1.08001656	3.00504593	1.645025	1.1300175	1.180018	2.01818976
Anti Mitochondria antibodies M4	2.03071694	1.630025	1.41074866	1.79454391	0.81501251	1.52502335	1.79502747	1.640025	1.31002	1.570024	3.94110033
HLA–C*08:02	22.3942924	15.722608	12.8028421	2.72463095	3.32735478	9.83485254	14.18481	42.8294895	12.903613	20.2486708	2.39082733
glucose transporter 2	2.97781599	1.230019	9.91602695	4.4501278	0.78501205	1.4300219	3.32505081	2.8350435	0.9300145	1.175018	1.73668256
HLA–C*17:01	13.0620432	3.37218864	5.74207696	2.55837718	2.53811633	3.77170595	2.82527975	3.97114999	3.01005378	4.63014446	3.04768819
Sm/RNP	1.46757705	0.910014	9.19385705	4.0323955	0.67501038	1.19501831	2.07503174	1.6700255	1.100017	1.7450265	1.22996961
Human KRT6A / CK6A / Cytokeratin 6A Protein	2.03006984	3.34304693	2.8289207	2.2296348	2.31133952	3.31876201	2.6702072	2.43743037	4.99704321	3.9308266	2.17430957
Recombinant Human CRMP5 Protein	3.14032617	4.6917375	4.92109589	3.21789566	1.44468312	13.792671	9.55229142	4.89426142	4.18339583	27.6045875	3.02921292
HA (Tyrosyl tRNA Synthetase)	42.7602276	5.97009	7.31286124	21.4791128	1.19001823	3.84505875	4.88507462	10.050155	1.3300205	4.2100645	16.2884358
AQP4 Recombinant	4.13601243	11.1001708	6.80232545	4.07899626	1.93340499	12.2138548	9.47026941	6.28587748	3.48338667	2.87087708	1.59169083
Platelet Glycoprotein	11.2073378	3.135048	0.69817674	11.4961634	1.72002632	3.17504852	7.04510758	3.480053	2.035031	2.4650375	1.30792555
ß2-Glycoprotein 1 (Apolipoprotein H)	25.6868613	5.515085	12.3392506	15.3282178	1.21001854	2.81504303	9.42514389	11.74518	1.575024	3.065047	7.80424689
Recombinant Human Galectin 3 Protein	3.46843759	3.83339208	5.0041511	2.94084591	2.51611173	4.7540163	2.75974148	4.06155685	4.89590833	2.28336833	1.6791925
Recombinant Human CDR2 Protein	2.73274277	22.1253375	4.90863761	2.3947021	3.86949522	5.66766953	7.50854567	3.17975937	3.57088792	3.57922125	2.92921125
Dopamine Receptor D1 (DRD1) Protein	7.48751041	2.45420417	4.44767289	2.5	1.93340499	5.18323415	59.9367952	2.5	3.77089083	2.89171083	1.82919458
Collagen Complex - 1	3.12713326	4.7500725	1.42514407	2.86018753	0.93501434	1.69502594	3.22004921	2.040031	1.2700195	1.505023	1.52013887
Cytochrome P450 2D6 (LKM 1 hp)	8.52815936	2.415037	2.43042123	1.43648766	0.84501297	1.22501877	2.18003334	2.750042	1.235019	1.225019	1.57210949
ASCA	3.11433482	1.4050215	6.45393478	14.2199485	0.83001274	1.21001854	1.93002953	2.940045	1.1300175	1.175018	6.01125985
TRP1 Protein	24.2150194	2.305035	1.85460658	6.94799643	0.64000984	1.14501755	2.38503647	9.525145	0.8150125	1.3400205	0.51537458
alpha-2B Adrenergic R/ADRA2B Recombinant Protein Antigen	1.46571306	1.204185	1.75664467	1.87710569	1.36143949	1.18975895	1.39952185	2.12424472	1.12918417	0.90834708	0.76251167
SS-A 60kDa	2.24829383	1.2850195	4.3905953	4.29241254	1.01501556	2.1250325	2.20003365	2.430037	1.245019	2.62004	1.77566075
FHL1 Protein	20.5716747	24.055365	1.44913627	7.52344398	0.73001122	5.04507706	5.4150827	20.035305	1.30002	1.2700195	0.82286709
Recombinant Endothelin Receptor A (ETRA)	4.33015339	47.279875	10.1287364	6.21115513	3.27604831	7.40712843	6.02529217	13.549852	5.95009167	5.09174583	7.67511667
Caspr2 Recombinant Protein Antigen	5.1271563	2.35003583	1.51162806	2.47519647	2.78195542	2.64558212	33.8294639	2.44335433	1.07084958	0.96668167	1.2583525

Parietal Cell (H/K-ATPase-α, βsubunits) (PCA)	8.35750923	1.705026	1.39635325	1.53026429	1.12001717	1.11501709	5.74008766	1.435022	1.1950185	4.330066	5.68644452
Ku(p70/p80)	1.03668942	0.855013	3.18857906	1.35123617	0.69501068	1.08501663	2.37503632	1.380021	0.9300145	2.1300325	1.71935931
SAE1/SAE2 Human Recombinant	27.9820824	2.230034	1.6026872	4.96163673	0.8800135	1.240019	4.45006798	7.71512	0.905014	1.4050215	0.73624953
Human Keratin 18 (KRT 18) Protein	1.7921932	1.82132811	1.6225433	1.89067036	1.92825119	1.79235045	1.64099915	1.89618973	2.03666461	1.88967473	1.86278049
HLA–C*07:02	25.3074051	12.0553661	23.8718359	2.87205362	4.26137129	10.9908933	12.8900764	44.0315547	11.2383219	19.9886394	6.42700382
INS-IGF2	21.3907877	6.965105	3.57245757	25.3239549	1.7250264	5.03507691	15.8002412	9.69015	3.1750485	5.29008	2.34300596
Human KRT10 / CK10 / Cytokeratin 10 Protein	10.9726955	1.640025	0.7797506	1.33418587	0.82501266	1.23501892	1.96503006	2.885044	1.170018	1.095017	0.81420547
HLA–A*33:01	1.31643921	1.93516027	1.40393053	1.11481767	1.66431799	1.74620543	1.33616572	1.60399471	1.68775919	1.60944166	1.97515039
Sm	1.95819117	1.120017	6.13243776	10.899403	0.78001198	1.59002434	3.88505936	1.770027	0.990015	2.560039	3.43005656
Tyrosinase Recombinant Protein Antigen	31.6040963	5.510085	1.07725542	25.0682004	2.26003456	11.2051711	124.716903	7.510115	3.530054	8.830135	6.76050355
Human MAG/Siglec-4a Protein	4.03950847	3.32505083	15.7931889	2.89872703	2.22073036	1.24246999	1.92241983	4.94146614	2.70420792	5.20424583	1.65002542
Muscarinic M2 Acetylcholine receptor	1.90699655	1.2750195	0.95969291	1.44501281	1.33502045	1.1350174	1.29501984	1.250019	0.985015	1.575024	2.8237336
Ribosomal P0	1.2627986	1.29002	1.02927079	2.22080136	0.76001167	1.32002022	2.04503128	1.780027	0.8650135	1.705026	1.13902123
HLA–B*07:02	1.87972063	2.39140317	2.02166072	2.02733241	2.08584264	2.23315129	2.14445093	2.01578104	2.07096451	1.83539507	3.46976006
Beta1 Adrenergic Receptor	8.08020543	2.0050305	0.50623797	1.21483379	0.78001198	1.10001686	1.50002296	2.435037	1.30502	1.685026	0.77522728
Glycine Receptor Alpha 1 Recombinant Protein	7.81221475	2.8833775	1.56561478	1.3992887	4.45757165	3.76757051	28.482569	2.43391338	0.95418125	0.89168042	2.41670375
HMGCR	3.01194559	1.0700165	1.58589259	2.30179027	0.77001183	1.02501572	1.17001793	1.430022	1.0700165	1.97503	4.88090025
Recombinant Human PNMA1 Protein	2.98478588	5.970925	7.2799012	4.08086819	2.92159078	8.17520154	8.05878517	4.09365606	3.03337958	2.54587208	4.55840417
Recombinant Human PNMA2 Protein	2.39554884	2.44170417	2.44186052	1.71284155	2.69065445	1.94779131	2.99555817	3.17787118	1.43335542	1.14168417	1.204185
Recombinant Human BACE-1 Protein	3.58310544	4.045895	4.53903778	3.40228351	2.59667038	3.6244979	6.09022626	2.91163275	7.004275	3.44588583	1.73752667
Fibrillarin	2.68344718	1.190018	3.36852114	16.9437335	0.89001366	1.31502014	1.72002632	3.3800515	1.7200265	2.540039	5.03248305
GAD II (GAD 65 kD)	1.24573401	1.385021	2.06573916	1.53026429	0.83001274	1.30001991	1.78502731	1.2200185	1.1400175	2.025031	1.45084455
Collagen Complex - 3	3.70733816	2.7750425	0.9237045	1.10400685	0.68501053	1.10001686	2.03503113	1.9350295	0.8100125	1.2050185	1.03941122
Tropomyosin	35.4522203	15.80024	3.45249539	28.2097178	1.01001549	6.30509628	24.1903692	15.77024	1.8500285	4.58007	9.56258309
ZO (Phenylalanyl tRNA Synthetase)	1.42918088	1.0900165	1.94097907	1.83716966	0.8600132	1.34002052	1.21001854	1.620025	1.055016	1.560024	2.42096147
KRT14 Human	35.4180911	3.3600515	1.06046081	11.2745095	0.97501495	2.7800425	9.48514481	15.515235	1.2200185	2.030031	0.85751403
SmD	1.31825936	0.9400145	12.555183	1.99062234	0.74501144	1.23001884	2.52003853	1.830028	1.105017	1.890029	3.12689487
SmD1	0.94283291	0.775012	16.6242813	1.61977836	0.60000923	0.82501266	1.89002892	1.0850165	0.925014	1.6050245	5.17539989
Recombinant Human SNCA	1.62465929	1.00834875	1.66943504	1.07918364	1.32921603	0.97389553	3.01264608	1.31797613	1.43335542	0.80834583	0.86251333
Recombinant Human Mannan Binding Lectin/MBL Protein	4.1984547	3.28755042	5.46926858	3.88197289	3.2626221	3.12751	3.51162013	4.32590709	2.78337583	5.34591667	1.65835875
21 Hydroxylase	5.08532357	2.095032	0.85892525	5.78857618	1.22001869	3.75505737	10.3451579	2.1900335	2.1350325	3.27505	2.01385895
Human KCNA1 Protein	3.34468609	5.30424583	6.88538066	4.35136619	3.49892622	7.55271104	5.59296101	7.33950205	5.7500875	2.41253708	1.74586
Human PLP1 / Myelin PLP Protein Recombinant	3.80335976	6.57926667	9.05315484	3.58667136	5.918368838	6.48343268	4.71633714	9.61480567	4.62507083	12.4085208	2.733375
Recombinant Human Neurofascin Protein	2.36603068	3.12088083	3.97009963	3.08451883	3.12298674	2.30421684	3.20403418	4.32968346	2.60837333	3.46671958	2.33336917
CENPA	1.42918088	1.9150295	6.57629616	1.20630864	1.09501678	1.64502518	4.40006722	2.215034	1.1300175	3.135048	2.91468198
Recombinant Human GRP78/HSPA5 Protein	3.16303359	3.07504708	4.71760648	1.9412202	2.959918419	3.17520004	4.10970751	2.95128472	3.63338875	2.20003375	1.73336
Glutamate Decarboxylase -GAD I (GAD 67 kD)	1.70221853	1.3450205	3.99472081	3.78942876	0.81501251	1.29501984	2.07503174	1.5350235	1.4000215	2.740042	4.84625374
F- actin	1.36092168	1.180018	4.74088267	1.24893438	1.08001656	1.16501785	1.30001991	1.4000215	1.1450175	1.94503	1.92724138
Asparaginyl-tRNA Synthetase (KS)	0.94283291	1.97003	3.70921412	6.00596747	0.95001457	1.39502136	2.22503403	1.0850165	0.990015	3.0300465	3.26115224
Oncomodulin Recombinant Protein	201.63396	13.75521	0.59500965	13.934356	1.90002907	4.53506928	101.756553	16.22525	1.710026	2.3750365	0.97011691
Nuclear Matrix Protein 2 (NXP2)	1.85153579	0.990015	8.99712061	3.0306905	0.69001061	1.10501694	1.67002556	2.2400345	1.035016	2.1850335	4.11000465
Human SEPT5 / Septin 5 Protein	13.0177032	33.217175	16.6694338	11.8831899	3.03168577	1.16967869	5.6151753	31.1480378	37.725575	3.67505625	4.20839583
Human PMEL/SILV/gp100 protein	3.28015799	5.26454745	6.65162378	3.67427037	5.27994897	5.67091696	2.70419607	3.79607931	3.40508624	13.8197477	5.082221
SS-B (La)	2.43600684	1.4000215	3.79798436	1.913896	0.8800135	1.31002007	3.40505203	1.555024	1.2600195	2.5300385	2.01385895
Human Melan-A/MART-1 Protein	29.6587036	10.45516	8.97792704	8.45268521	1.04501602	4.79507324	8.95513672	32.16549	3.1700485	1.845028	18.5361626
Glycyl-tRNA Synthetase (EJ)	2.77730369	3.220049	2.24328228	2.23785165	0.88501358	1.23501892	1.84502823	1.500023	1.1450175	2.155033	3.0619318
Mi-2	0.83617754	1.2100185	12.4304224	1.51747657	0.95501465	1.09001671	1.77502716	1.7300265	0.9600145	2.3350355	5.0151598
Human EEF1A1 Recombinant Protein	1.53269772	1.82086125	1.43272437	1.57759231	1.58700318	2.10090368	2.29323277	2.44524252	2.4625375	0.82084583	0.975015
SUMO	44.8336175	11.03517	2.80470295	18.1457795	1.05501617	3.76005745	8.68013252	38.900595	2.285035	1.8050275	0.92247753
Scl-70	1.26706475	1.1950185	4.35220817	1.78601876	0.82501266	1.31002007	1.78502731	1.8100275	0.9400145	2.61504	2.76743216
Recombinant Human Beta-amyloid 42	4.64463991	4.46673333	4.90033209	4.6710028	2.33619772	1.40311237	2.98530542	4.66389858	5.2209125	2.52920542	1.94169625
SS-A 52kDa (Ro-52)	2.06484654	2.290035	5.96929006	3.52088657	1.26501938	2.12003243	3.31005058	2.415037	2.2500345	3.0500465	2.24339552
SLA (Soluble Liver Antigen)	2.35921535	1.115017	1.71305188	3.06052852	0.9650148	1.02001564	1.48002266	1.2850195	1.165018	1.7400265	3.72022539
BCOADC-E2	1.99232077	1.4100215	1.55950095	5.68201182	0.87001335	1.21501862	3.26004982	1.4850225	1.3950215	1.375021	1.90558688
Recombinant Human HOMER3 protein	4.16439528	7.8167875	6.79401993	3.87495269	2.55370568	1.31777105	3.25529794	3.14010551	8.7168	2.87504375	1.95419667
Islet Cell Antigen (IA-2)	3.61348123	1.7350265	0.5542226	1.41091221	0.82001259	1.38502121	1.81502777	2.2050335	1.045016	1.2650195	0.95712447
HLA–B*42:01	2.26582892	2.01620993	2.22121943	2.16237983	1.69250462	1.86520909	2.08709545	1.89865671	1.82968726	2.38197477	1.81070656
Alanyl-tRNA Synthetase (PL-12)	1.72354969	1.8500285	1.41074866	3.2821824	1.57002403	1.48502274	1.88002876	1.4750225	1.4100215	1.6500255	3.63793864
Recombinant Human TRP2 Protein	27.9266224	11.15517	0.68378133	4.19011076	1.77002708	10.3901586	27.0854134	13.86021	2.3750365	8.62513	2.32568227
Collagen Complex - 2	2.89675793	2.080032	0.55182341	2.19948848	0.80001228	1.12501724	1.94502976	2.480038	1.180018	1.5500235	1.36422699
Recombinant Human GABA Receptor Epsilon (gABRe)	2.32742887	7.27094583	2.02242507	2.86362742	2.5	10.9663663	3.11688409	11.805137	1.51252333	1.68335917	2.55003917
Recombinant Human Glutamate Receptor 3 (GRIA3)	5.17597594	8.10429167	7.50830718	4.54792158	2.65306131	6.1219874	14.287427	7.03361165	2.79170917	5.0709125	1.61252458
Human OMGP/OMG Recombinant Protein	2.47956321	2.86671042	3.93687713	3.42147125	2.42749735	2.5	2.57006217	4.39388378	2.1250325	5.10424583	1.2458525
Human Cytochrome P450 1B1 (CYP1B1) Protein	1.47031841	1.93060702	2.08483765	2.36683488	2.03331173	3.2847601	1.54328209	2.05893166	2.18568897	2.08785653	2.80193646
SmD3	9.15529145	1.9300295	2.21209226	1.50042627	0.96001472	1.56002388	4.07506226	2.095032	1.780027	1.6050245	3.6032917
Cardiolipin	3.25938594	1.4600225	4.15546932	2.71099742	2.08003182	5.37008202	5.500084	1.9300295	1.1350175	3.740057	2.71979235
CYP21A2	108.203941	9.18014	1.15643008	10.2642794	1.00001534	1.80502762	69.2160562	13.565205	1.905029	1.225019	4.94586245
Pancreatic and duodenal homeobox 1(PDX1)	8.11433461	2.225034	0.94049911	9.95311142	1.23501892	1.90002907	7.2301104	4.110063	2.740042	2.6550405	2.08315326
Recombinant Human Acetylcholine receptor	3.50817358	5.82925417	7.14285596	3.28388211	2.08378105	3.11244999	3.50307617	5.47583118	6.3542625	2.79170917	1.75002667
Threonyl-tRNA Synthetase(PI-7)	2.69624605	1.3450205	3.27255331	1.57715261	1.22501877	1.41502167	2.77004234	2.7050415	1.0850165	1.4650225	1.2472933
Recombinant Dipeptidyl Peptidase 6 (DPP6)	3.4071295	3.59588833	2.36295683	2.33152355	3.49087049	4.45532106	1.73103173	8.86707149	1.90002917	2.66254083	2.13336583
SP100	1.93686001	1.240019	0.8205374	1.07416883	0.70001076	1.14501755	1.12001717	1.4600225	0.965015	1.250019	1.09138185
Human Involucrin (iNV) Protein	2.16841953	1.49530997	1.70076211	1.65769912	1.73222343	1.97571379	2.04673387	2.02441117	1.78829108	1.9868722	1.20683319
SRP54	0.81058023	2.275035	7.64875343	2.17391304	0.95501465	1.23501892	2.65504059	1.110017	1.0650165	2.61004	4.55175411
Ganglioside GM1	2.18891829	1.75419333	1.27906974	1.33845006	3.46133281	1.64407632	3.25017156	0.96110293	3.95839375	0.85001292	0.97918167
OJ (Isoleucyl tRNA Synthetase)	4.87627812	1.430022	2.94865709	5.52429656	1.16001778	2.17003319	2.91004448	3.150048	0.9950155	2.3850365	5.30099781
OGDC-E2	1.36518783	1.31002	0.87092147	2.03751066	0.74001137	1.08001656	1.44502213	1.105017	1.0600165	1.29002	1.33824168
Insulin	1.84726965	1.3250205	2.92706433	1.11679457	0.7750119	0.99001518	1.67502564	1.5400235	1.105017	1.6050245	1.66305787
MDA5	1.11774748	1.125017	5.99088282	1.82011936	0.7550116	1.28001961	1.86502853	2.345036	1.0900165	2.370036	2.64616722
Intrinsic factor	1.61262817	1.2000185	4.98560543	2.60869563	1.05501617	1.07501648	1.74002663	2.560039	1.185018	2.4000365	2.3776529
Zinc transporter 8	6.26279711	1.7300265	7.00575939	1.6794544	1.09501678	1.86002846	8.21012535	1.775027	1.29502	1.845028	2.81940236
Alpha-Myosin	3.14846442	1.1450175	1.52111334	3.55072459	1.02501572	1.2600193	3.82505844	1.4550225	1.235019	1.625025	1.45517537
Myeloperoxidase	2.07337883	1.29502	4.37380093	1.57289004	0.90001381	1.27001946	2.65004051	1.190018	1.125017	2.095032	2.61152028
PM/SCl100	1.72354969	1.565024	9.74088327	1.67092925	0.92001411	1.34002052	1.75002678	1.7950275	1.185018	4.0250615	5.11909932
Recombinant Inositol-1,4,5-Trisphosphate Receptor Type 3 (ITPR3)	2.01180704	1.46668917	1.8646181	1.46574313	2.10526327	2.44979921	2.50854396	3.99358016	1.01251542	0.975015	0.92501417
Th/To antigen	2.06058039	1.125017	2.4424196	2.71099742	0.81501251	1.08501663	1.51502319	1.3350205	1.095017	1.5850245	3.01429199
Glomerular Basement Membrane	0.99829367	1.2150185	2.95585468	2.90707584	0.82001259	1.18001808	1.61502472	1.245019	0.8100125	1.705026	3.02728486

Table S4. A panel of Neuronal Zoomer antigens used to measure plasma antibody levels via protein microarray in Mn-exposed workers and control groups.

Neuonal Zoomer Antigen	3000/2675	3001/2849	3002	3003	3004/4755	3005/5608	3006/5965	3007/6013	3008/5997	3009/6069	3010/6166
Group	Workers	Workers	Workers	Workers	Workers	Workers	Controls	Controls	Controls	Controls	Controls
Recombinant Human CRMP5 Protein	3.14032617	4.6917375	4.92109589	3.21789566	1.44468312	13.792671	9.55229142	4.89426142	4.18339583	27.6045875	3.02921292
Human SEPT5 / Septin 5 Protein	13.0177032	33.217175	16.6694338	11.8831899	3.03168577	1.16967869	5.6151753	31.1480378	37.725575	3.67505625	4.20839583
Recombinant Calcium Channel Alpha 1B Subunit (CACNa1B)	4.93528483	25.7003917	23.1021594	4.81233721	4.70461944	6.90010052	4.90430766	6.06117921	10.404325	3.25838292	3.57922125
Recombinant Endothelin Receptor A (ETRA)	4.33015339	47.279875	10.1287364	6.21115513	3.27604831	7.40712843	6.02529217	13.549852	5.95009167	5.09174583	7.67511667
AQP4 Recombinant	4.13601243	11.1001708	6.80232545	4.07899626	1.93340499	12.2138548	9.47026941	6.28587748	3.48338667	2.87087708	1.59169083
Glycine Receptor Alpha 1 Recombinant Protein	7.81221475	2.8833775	1.56561478	1.3992887	4.45757165	3.76757051	28.482569	2.43391338	0.95418125	0.89168042	2.41670375
Recombinant Human NARG2 Protein (NMDA)	5.26907282	4.2959	5.71843836	2.98717663	4.31525373	5.10793159	2.15652772	6.02152724	3.66255583	1.78336042	1.80419417
Dopamine Receptor D1 (DRD1) Protein	7.48751041	2.45420417	4.44767289	2.5	1.93340499	5.18323415	59.9367952	2.5	3.77089083	2.89171083	1.82919458
Caspr2 Recombinant Protein Antigen	5.1271563	2.35003583	1.51162806	2.47519647	2.78195542	2.64558212	33.8294639	2.44335433	1.07084958	0.96668167	1.2583525
Recombinant Human GABA Receptor Epsilon (gABRe)	2.32742887	7.27094583	2.02242507	2.86362742	2.5	10.9663663	3.11688409	11.805137	1.51252333	1.68335917	2.55003917
Recombinant Human GFAP Protein	1.53496835	1.48335583	1.87707638	1.78491176	2.20730414	2.36194789	1.94292533	1.42749279	1.16251792	1.0416825	1.09168333
Recombinant Human HOMER3 protein	4.16439528	7.8167875	6.79401993	3.87495269	2.55370568	1.31777105	3.25529794	3.14010551	8.7168	2.87504375	1.95419667
Human PLP1 / Myelin PLP Protein Recombinant	3.80335976	6.57926667	9.05315484	3.58667136	5.91836838	6.48343268	4.71633714	9.61480567	4.62507083	12.4085208	2.733375
Recombinant Dipeptidyl Peptidase 6 (DPP6)	3.4071295	3.59588833	2.36295683	2.33152355	3.49087049	4.45532106	1.73103173	8.86707149	1.90002917	2.66254083	2.13336583
Recombinant Human Glutamate Receptor 3 (GRIA3)	5.17597594	8.10429167	7.50830718	4.54792158	2.65306131	6.1219874	14.287427	7.03361165	2.79170917	5.0709125	1.61252458
Recombinant Human PNMA1 Protein	2.98478588	5.970925	7.2799012	4.08086819	2.92159078	8.17520154	8.05878517	4.09365606	3.03337958	2.54587208	4.55840417
Recombinant Human GRP78/HSPA5 Protein	3.16303359	3.07504708	4.71760648	1.9412202	2.95918419	3.17520004	4.10970751	2.95128472	3.63338875	2.20003375	1.73336
Human KCNA1 Protein	3.34468609	5.30424583	6.88538066	4.35136619	3.49892622	7.55271104	5.59296101	7.33950205	5.7500875	2.41253708	1.74586
Cluster of differentiation 11b (CD11b)	2.46593945	1.86252833	1.66528227	1.54483308	2.05692808	2.86646479	2.22146352	2.61706961	1.25418583	0.93751417	1.10001667
Recombinant GRB2 Related Adaptor Protein 2 (GRAP2)	3.31743856	2.67087417	1.83139518	1.95993996	2.93233175	9.22439738	1.35680137	3.73489449	1.74586	1.23751875	1.07918333
Recombinant Human AIF-1/Iba1 Protein	3.30267834	3.95006042	5.20764051	3.87588865	2.65843179	3.36596269	4.01913984	2.65483339	3.55005417	3.06671333	1.27501958
Recombinant Inositol-1,4,5-Trisphosphate Receptor Type 3 (ITPR3)	2.01180704	1.46668917	1.8646181	1.46574313	2.10526327	2.44979921	2.50854396	3.99358016	1.01251542	0.975015	0.92501417
Recombinant Human GAD2/GAD65 Protein	3.12897303	3.14171458	1.8978406	3.25346279	4.59452176	5.01254901	2.5	4.78096819	1.30418667	0.97918167	1.61252458
Recombinant Adaptor Related Protein Complex 3 Beta 2 (AP3b2)	2.02202486	1.98336375	1.92691034	1.85885438	2.55907644	2.16616473	1.38926892	2.16389858	1.27918625	0.86668	1.08335
Human MAG/Siglec-4a Protein	4.03950847	3.32505083	15.7931889	2.89872703	2.22073036	1.24246999	1.92241983	4.94146614	2.70420792	5.20424583	1.65002542
Recombinant Human Amphiphysin/AMPH Protein	1.96071677	2.67504083	5.0830577	2.01656676	2.72287737	3.45883527	1.23376648	2.33761386	1.39168792	1.98753042	1.30835333
Recombinant Human Dopamine D2R/DRD2	2.51930034	3.75422417	3.4260797	3.90256411	2.98066614	2.07831316	1.33629552	4.24848945	2.25003458	1.51252333	1.30418667
Recombinant Human CDR2 Protein	2.73274277	22.1253375	4.90863761	2.3947021	3.86949522	5.66766953	7.50854567	3.17975937	3.57088792	3.57922125	2.92921125
Human EEF1A1 Recombinant Protein	1.53269772	1.82086125	1.43272437	1.57759231	1.58700318	2.10090368	2.29323277	2.44524252	2.4625375	0.82084583	0.975015
Recombinant Human PNMA2 Protein	2.39554884	2.44170417	2.44186052	1.71284155	2.69065445	1.94779131	2.99555817	3.17787118	1.43335542	1.14168417	1.204185
Human NOVA1 Protein	1.8358311	1.8958625	2.41279078	2.36568673	2.39795913	3.04969994	1.85406815	1.36329323	1.51252333	1.80419417	1.67085875
Recombinant Human Titin protein	2.54541256	2.27503458	4.52242673	2.8818792	2.53759395	10.4216858	3.18352697	2.39614772	1.93752958	1.35002083	2.52920542
Recombinant Human S100B	3.73410447	66.226	4.64285679	6.88459106	2.8974209	2.85391478	3.46377396	3.06835433	3.48338667	2.97921208	1.63335833
Recombinant Human SNCA	1.62465929	1.00834875	1.66943504	1.07918364	1.32921603	0.97389553	3.01264608	1.31797613	1.43335542	0.80834583	0.86251333
Recombinant Human CD68/SR-D1 Protein	2.75544905	1.75419333	2.75747489	2.98951654	1.16272824	1.29769079	0.87662352	2.41691968	2.44170417	1.37085417	0.65834333
Recombinant Human BACE-1 Protein	3.58310544	4.045895	4.53903778	3.40228351	2.59667038	3.6244979	6.09022626	2.91163275	7.004275	3.44588583	1.73752667
Human Tau-441 / 2N4R Protein	6.41008041	5.02090833	4.99999834	4.70656853	2.68528396	4.6234937	3.7491456	4.37311181	4.62090417	4.2334	1.56669083
Recombinant Human Enolase 2/Neuron-specific Enolase Ptotein	4.02247876	4.4709	6.96428726	4.05934053	3.44253611	3.05973995	2.8827762	3.20052945	4.6667375	2.01253083	3.57922125
Ganglioside GM1	2.18891829	1.75419333	1.27906974	1.33845006	3.46133281	1.64407632	3.25017156	0.96110293	3.95839375	0.85001292	0.97918167
Recombinant Bone Morphogenetic Protein 1 (BMP1)	3.09037236	1.9208625	1.85631216	0.52087229	3.47475903	2.02058237	1.48325401	1.46336876	1.47085583	0.92501417	1.29168625
Recombinant Leucine-rich glioma-inactivated protein 1 (LGI1)	2.70322347	2.1375325	1.76079743	1.18214163	1.78571444	1.75702816	1.28503109	2.03549795	1.00834875	0.88751375	0.84167958
5-Hydroxytryptamine (5-HT)	2.35808348	5.91259167	5.36544956	4.24372838	3.66272877	5.8659647	3.34073924	1.83345946	2.10419875	1.62919167	2.65420708
Recombinant Human MBP Protein	3.54677539	3.29588375	5.2200988	3.23895534	1.37218047	3.29568264	3.40909091	3.24962236	1.96669667	2.52087167	1.08335
rhCarbonic Anhydrase VIII	3.24477733	2.7750425	4.65116231	2.98109286	3.61976488	3.81275055	3.09637859	2.65105701	2.77920917	2.74170875	1.10835042
Recombinant Human Galectin 3 Protein	3.46843759	3.83339208	5.0041511	2.94084591	2.51611173	4.7540163	2.75974148	4.06155685	4.89590833	2.28336833	1.6791925
Recombinant Human Mannan Binding Lectin/MBL Protein	4.1984547	3.28755042	5.46926858	3.88197289	3.2626221	3.12751	3.51162013	4.32590709	2.78337583	5.34591667	1.65835875
POLY(L-GLUTAMIC ACID)	1.33742031	1.65835875	1.59883728	1.02957705	1.726638	2.24146579	1.42686303	1.16125398	1.17918458	0.88751375	1.60835792
Human GRM1 / MGLUR1 Protein	1.65077151	1.71252625	1.47425239	1.78023194	3.40494001	2.67319214	1.81134665	2.03738614	1.33335375	0.92918083	1.14168417
Tubulin Beta (TUBb)	1.84831955	2.00003042	2.5	2.5290149	2.88130944	3.31325265	1.82843457	2.08081638	1.45835583	1.18335125	1.31668667
Recombinant Human RAGE Protein	2.10830986	1.42918833	1.71511623	1.33470621	1.11439305	1.566265	0.73479175	1.73904869	0.87084667	0.98334833	0.95001458
alpha-2B Adrenergic R/ADRA2B Recombinant Protein Antigen	1.46571306	1.204185	1.75664467	1.87710569	1.36143949	1.18975895	1.39952185	2.12424472	1.12918417	0.90834708	0.76251167
Human Brain Cerebellum Protein	1.25681188	1.36252083	2.03073101	1.17512142	2.33351247	1.45080316	1.35167499	1.11216031	0.89168042	0.975015	0.88751375
GM2	1.74613902	1.23335208	1.85631216	1.95385619	1.80451141	1.76957842	1.29186642	1.15181285	0.770845	0.81667917	0.758345
Human DBH/Dopamine beta-Hydroxylase Protein	1.79609397	1.66669208	1.94352179	2.39563807	2.76047347	3.30321264	1.37388963	1.82401852	1.21251875	1.07501667	1.05001625
Beta-Amyloid (25-35)	1.75635799	2.42920375	2.16777418	3.01151218	1.97099894	2.16365473	1.08168179	1.44637487	1.38335458	1.19585167	0.92501417
Human STK23/MSSK1/SRPK3/SPRY3 recombinant protein	2.36262474	2.26253458	3.90780739	2.73633435	1.57626221	1.89759052	2.08304967	1.5143508	1.97503	1.95836333	1.2458525
Recombinant Human Acetylcholine receptor	3.50817358	5.82925417	7.14285596	3.28388211	2.08378105	3.11244999	3.50307617	5.47583118	6.3542625	2.79170917	1.75002667
Recombinant Human Beta-amyloid 42	4.64463991	4.46673333	4.90033209	4.6710028	2.33619772	1.40311237	2.98530542	4.66389858	5.2209125	2.52920542	1.94169625
Recombinant Human Recoverin Protein	3.96003536	3.56255458	5.75996596	3.37981989	2.07572505	2.14859447	2.76828544	3.63481858	3.47088625	2.64587375	0.92918083
Human Siglec-1 / CD169 Protein	2.15485887	3.02921292	4.33554836	3.45797434	2.93233175	3.58433787	2.93062237	1.91465197	1.88752875	1.95003	1.21251875
Human ELAVL3 / HUC Protein	3.48319667	2.46670417	3.77491698	3.62457839	2.05692808	1.61646579	2.05912488	3.05136063	2.52087167	1.61252458	1.44168875
Recombinant Human NRXN3 Protein	2.5	3.32088417	4.70930095	4.18008185	2.75778823	2.93674735	2.90840808	3.57439653	1.96253	3.27921667	1.408355
Human OMGP/OMG Recombinant Protein	2.47956321	2.86671042	3.93687713	3.42147125	2.42749735	2.5	2.57006217	4.39388378	2.1250325	5.10424583	1.2458525
Recombinant Human Neurofascin Protein	2.36603068	3.12088083	3.97009963	3.08451883	3.12298674	2.30421684	3.20403418	4.32968346	2.60837333	3.46671958	2.33336917

Table S5. The 5 top enriched metabolite sets for ANCOVA results in metabolite enrichment analysis using RaMP-DB in plasma samples of Mn-exposed workers compared than Control group. Hits: The metabolites in the set that are significantly changed in the experimental data. P < 0.05, * FDR < 0.05.

Enriched Metabolite Sets	Hits
**Biogenic amine synthesis***	**Choline, Glutamic acid, Phenylalanine**
**Transmission across Chemical Synapses***	**Choline, Homovanillic acid, 3,4-Dihydroxybenzeneacetic acid, Glutamic acid**
**Neuronal System***	**Choline, Homovanillic acid, 3,4-Dihydroxybenzeneacetic acid, Glutamic acid**
**22q11.2 copy number variation syndrome***	**Homovanillic acid, 3,4-Dihydroxybenzeneacetic acid, Glutamic acid**
**Neurotransmitter clearance***	**Choline, Homovanillic acid, 3,4-Dihydroxybenzeneacetic acid**

Table S6. The 5 top enriched metabolite sets for linear regression results in metabolite enrichment analysis using RaMP-DB in plasma samples of Mn-exposed workers compared than Control group. Hits: The metabolites in the set that are significantly changed in the experimental data. P < 0.05.

Enriched Metabolite Set	Hits
**SLC-mediated transmembrane transport**	L-Carnitine, Citric acid, Hypoxanthine, L-Tyrosine, Proline, Lactic acid, Uracil, Creatinine, N-Acetylneuraminic acid, L-Aspartic acid, L-Valine, L-Threonine, 4-Hydroxyproline
**Transport of small molecules**	L-Carnitine, Citric acid, Hypoxanthine, L-Tyrosine, Proline, Lactic acid, Uracil, Creatinine, N-Acetylneuraminic acid, L-Aspartic acid, L-Valine, L-Threonine, 4-Hydroxyproline
**Biochemical pathways: part I**	Pipecolic acid, Citric acid, Hypoxanthine, L-Tyrosine; Proline, L-Threonine, Lactic acid, L-Aspartic acid, N6-Acetyl-L-lysine, Xanthine, Uracil, 4-Hydroxyproline, L-Valine; Citrulline; 5-Aminolevulinic acid, Methionine sulfoxide, Carglumic acid
**Transport of bile salts and organic acids, metal ions and amine compounds**	L-Carnitine, Citric acid, L-Tyrosine, Proline, Lactic acid, Creatinine, L-Valine, L-Threonine, 4-Hydroxyproline
**Transport of inorganic cations/anions and amino acids/oligopeptides**	L-Tyrosine, Proline, Lactic acid, N-Acetylneuraminic acid, L-Aspartic acid, L-Valine, L-Threonine, 4-Hydroxyproline

Table S7. Fold changes of significantly distinct antibodies. Fold changes in the antibodies (expressed in signal intensity unit or arbitrary units (AU)) targeting two panels of AI (Autoimmune) and NZ (neuronal Zoomer) proteins with significantly different abundance in the serum samples of Mn exposed workers identified using protein microarray. ** ($p < 0.01$) * ($p < 0.05$).

Antigen Name	Fold Change
5-Hydroxytryptamine (5-HT) (Serotonin)	1.97*
Beta-Amyloid (25-35)	1.86**
Recombinant Human Amphiphysin/AMPH Protein	1.81*
GM2	1.80**
Human DBH/Dopamine Beta-Hydroxylase Protein	1.77*
Human Siglec-1 / CD169 Protein	1.64*
Tubulin Beta (TUBb)	1.60*
Recombinant Adaptor Related Protein Complex 3 Beta 2 (AP3b2)	1.54*
rhCarbonic Anhydrase VIII	1.42*
Human NOVA1 Protein	1.42*
Recombinant Human GFAP Protein	1.41*
Anti Mitochondria antibodies M2	0.74*
